# The Association Between *Mycoplasma pneumoniae* and *Chlamydia pneumoniae*, a Life-Threatening Condition in Small Children—A Case Report and a Review of the Literature

**DOI:** 10.3389/fped.2020.558941

**Published:** 2020-11-10

**Authors:** Cristina Oana Mărginean, Lorena Elena Meliţ, Iunius Simu, Maria Oana Săsăran

**Affiliations:** ^1^Department of Pediatrics I, George Emil Palade University of Medicine, Pharmacy, Science, and Technology of Târgu Mureş, Târgu Mureş, Romania; ^2^Department of Radiology, George Emil Palade University of Medicine, Pharmacy, Science, and Technology of Târgu Mureş, Târgu Mureş, Romania; ^3^Department of Pediatrics III, George Emil Palade University of Medicine, Pharmacy, Science, and Technology of Târgu Mureş, Târgu Mureş, Romania

**Keywords:** *Mycoplasma pneumoniae*, *Chlamydia pneumoniae*, coinfection, pneumonia, child

## Abstract

*Mycoplasma pneumoniae* (MP) and *Chlamydia pneumoniae* (CP) are two atypical pathogens that may result in mild, moderate or severe acute respiratory infections. We report the case of a 2 years and 9-month-old male child admitted with prolonged fever, dry cough, and shortness of breath for which he underwent symptomatic treatment. The laboratory tests showed leukocytosis with neutrophilia, anemia, and elevated inflammatory biomarkers and the thoracic radiography revealed pleural effusion raising the suspicion of inferior right pneumonia. Although we the initial evolution was favorable being treated with 3rd class cephalosporin and Oxacillin, on the 8th day of admission the fever and the acute phase reactants levels increased as well as the quantity of the pleural effusion, requiring surgical drainage. We ruled out lung tuberculosis, but we identified positive IgM for both MP and CP. Based on these findings we changed the antibiotic therapy on Levofloxacin for 10 days with favorable evolution. MP and CP are two atypical pathogen that are difficult to be diagnosed due to their slow-growing pattern. Despite their self-limiting feature, the association between them might carry a vital risk in small children, especially in the lack of a proper and timely diagnosis.

## Introduction

Community-acquired pneumonia represents an acute infection of the lungs diagnosed in a previously healthy subject consisting in suggestive clinical symptoms and lung imaging (1). This condition is an important leading cause of death in children under the age of 5 years resulting in up to 2 million deaths each year (2). *Mycoplasma pneumoniae* (MP) and *Chlamydia pneumoniae* (CP) are two atypical pathogens that may result in mild, moderate or severe acute respiratory infections (3). The prevalence of infections caused by these two pathogens during childhood express a wide variation worldwide depending on season and geographic area (3). Thus, recent data underlined the highest incidence between 1 and 7 years of age, with a peak around the age of 5 years (4). The clinical spectrum of MP infection ranges from mild cases of tracheobronchitis to severe ones of atypical pneumonia associated with a wide spectrum of extrapulmonary complication (5), whereas CP, another pathogen involved in the development of community-acquired pneumonia might also cause pharyngitis, bronchitis and sinusitis (6). Despite its self-limiting pattern, MP infection might also rarely lead to life-threatening conditions such as acute respiratory distress, fulminant pneumonia, necrotizing pneumonitis or refractory pneumonia (7, 8). The incubation period for these group of fastidious, slow-growing pathogens that includes also Legionella pneumophila, reaches up to 3 weeks, defined by further unspecific clinical features such as cough, fever and malaise caused also by other microorganisms (9, 10). Moreover, both MP and CP were associated with the presence or exacerbation of asthma in children, and even wheezing in those without a previous diagnosis of asthma (11, 12).

It is a well-known fact that the main challenges related to the management of lower respiratory tract infections in children consists in determining the infecting pathogen. Despite their ubiquity, both MP and CP are usually underdiagnosed since they are difficult to grow in culture and they require specific culture techniques or they present a long period of incubation in order to confirm or exclude their presence (13). Thus, standardized, rapid and specific diagnostic tests would definitely improve their rate of detection in children leading to better outcomes in severe cases. Among these tests, serological ones or the detection of nucleic acid and DNA sequences are commonly used (14). Serological tests are very useful for the detection of these two atypical bacteria but their accurate interpretation might be hindered by the preexisting IgG antibodies in most of the people due to prior exposure (15). Nevertheless, positive IgM antibodies present certain utility for the diagnosis of acute phase infection in case of both MP and CP. Lung imaging consisting of chest radiography or thoracic computed tomography confirm the diagnosis of lung impairment showing different aspects varying from interstitial pneumonia to massive pneumonia with pleural effusion. Except for low yield of cultures in case of atypical bacteria, the difficulty of obtaining appropriate culture specimens and care-givers' reluctance to allow their children undergo lung aspiration and bronchoalveolar lavage contribute additionally to the challenges in establishing the appropriate diagnosis (16). Nevertheless, pediatrician's awareness regarding these atypical bacterial as causative agents in childhood community acquired pneumonia implying a thorough anamnesis and clinical exam (17) along with appropriate serological tests or polymerase chain reaction when available, definitely increase the rate of early diagnosis.

B-lactam antibiotics are usually the first choice in the treatment of lower respiratory tract infections being commonly used as empirical therapy, but their efficacy for MP and CP is either low or even absent. Even though most of the cases diagnosed with community acquired pneumonia caused by MP or CP infection are self-limiting, antibiotics such as macrolides, tetracyclines, or fluoroquinolones might be required for certain groups of patients (18). Therefore, the early diagnosis and appropriate detection of etiological agent taking into account these cases where antibiotics are of major importance, represent the factors that influence the outcome.

Our aim in presenting this case was to underline the evolution peculiarities, as well as diagnostic and treatment challenges in a small child with community acquired pneumonia caused by coinfection with MP and CP.

*The written informed consent was obtained from the patient's mother prior to publication of this case*.

## Case Report

### Presenting Concerns

We report the case of a 2 years and 9-month-old male child admitted in our clinic for prolonged fever, dry cough and shortness of breath. The disease onset was ~7 days before the admission with fever (39°C) and dry cough for which he was recommended symptomatic treatment by the general practitioner, but the symptoms worsened and thus he was admitted to our clinic.

### Clinical Findings

The clinical exam at the time of admission revealed influenced general status, pallor, hyperemic pharynx, diminish breath sounds on the inferior right thorax, rales on the right lung field, tachypnea (45/min), oxygen saturation 85%, and tachycardia (154 beats/min).

### Diagnostic Focus and Assessment

The laboratory tests performed on the day of admission showed leukocytosis (Leu 23,890/μL) with neutrophilia (Neu 20,430/μL), anemia (Hemoglobin 10 g/dL), and elevated inflammatory biomarkers (C-reactive protein- CRP 303 mg/L, erythrocyte sedimentation rate- ESR 100 mm/h). The blood culture was negative. The thoracic radiography revealed pleural effusion raising the suspicion of inferior right pneumonia.

### Therapeutic Focus and Assessment

**Figure 1:**
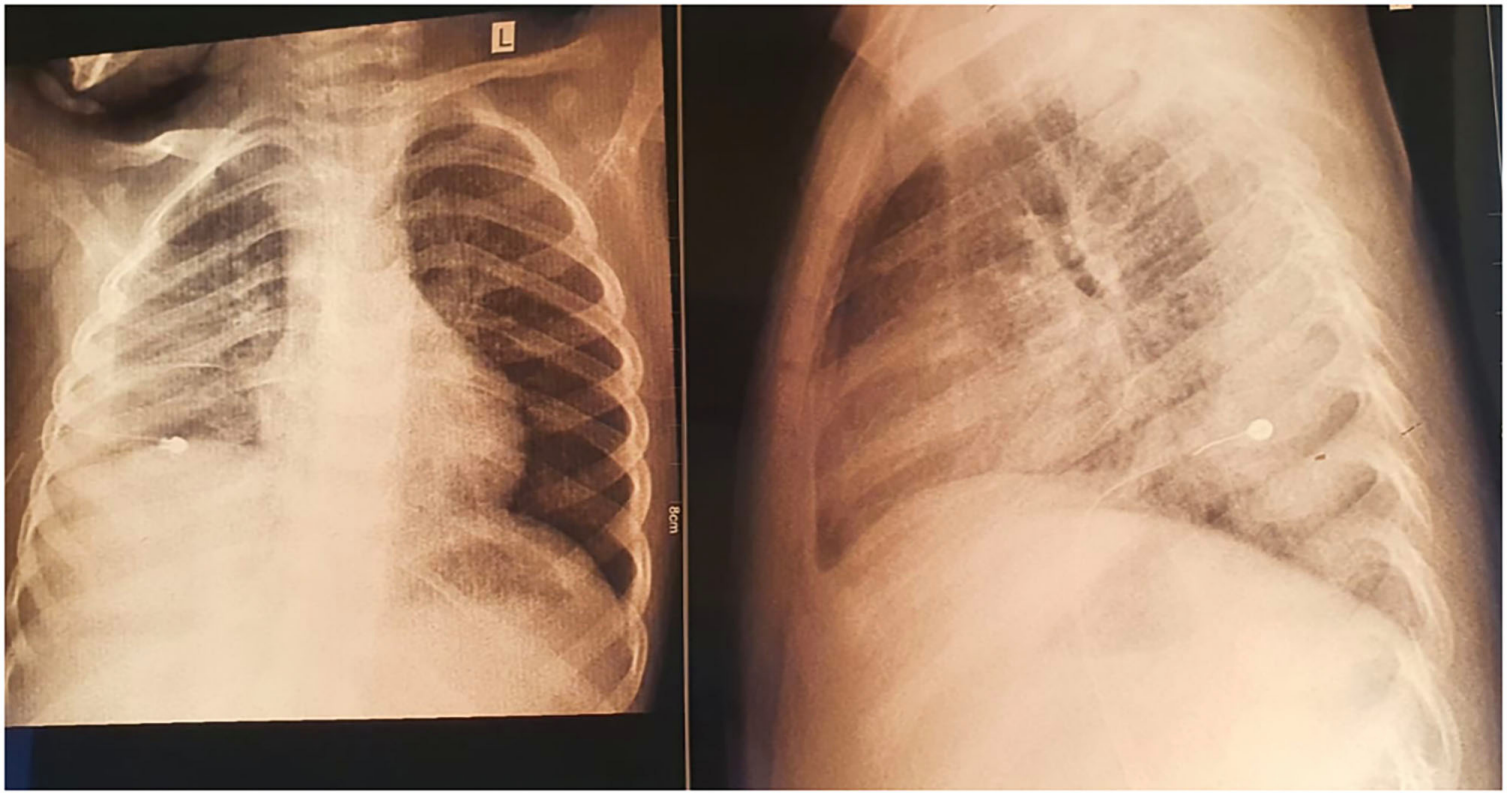
Thoracic radiography revealing right pleural effusion.

**Figure 2:**
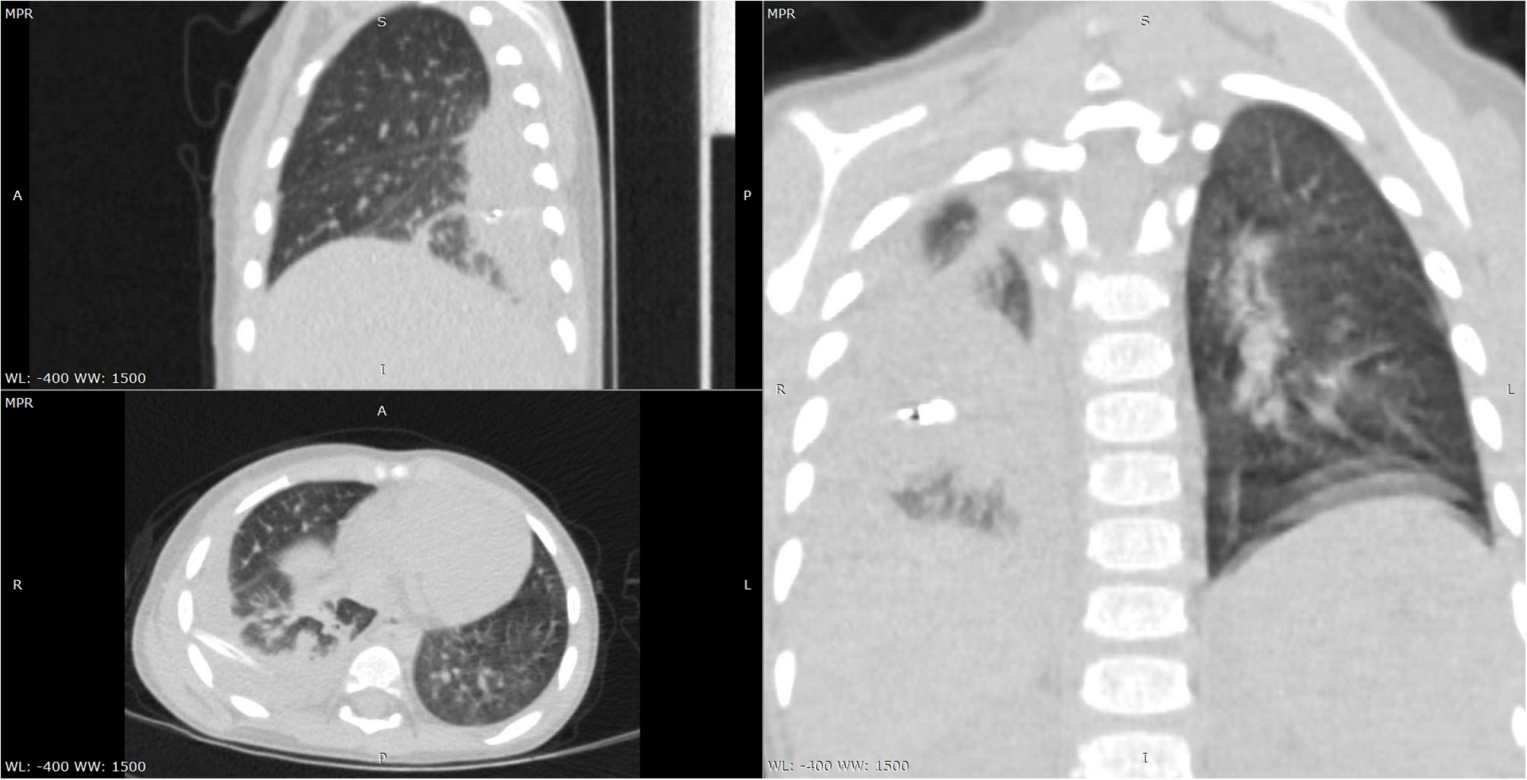
Chest CT scan—multiplanar reconstructions lung window-encapsulated pleural effusion with drain tube.

**Figure 3:**
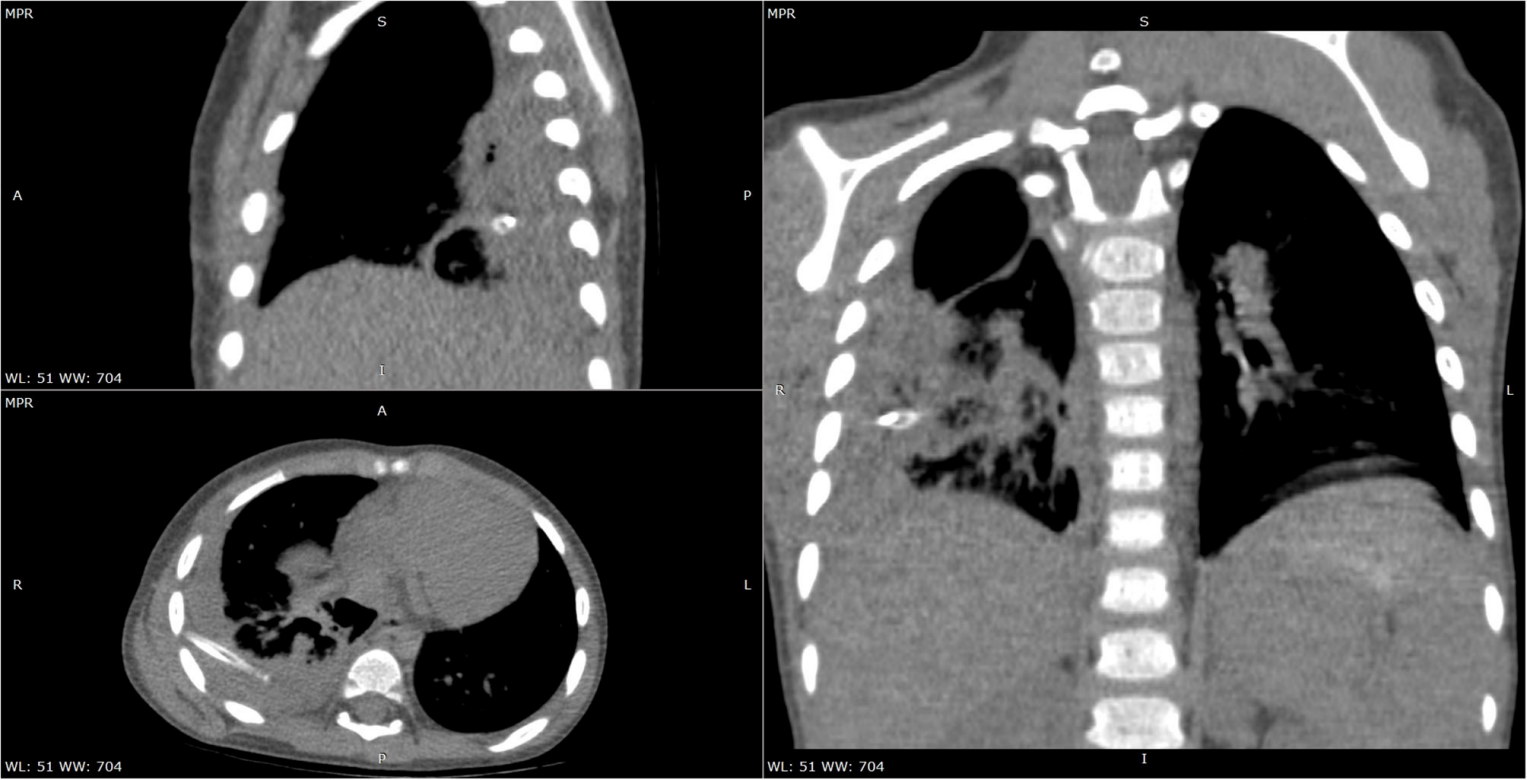
Chest CT scan—multiplanar reconstructions soft tissue window-encapsulated pleural effusion with drain tube.

We initiated empirical treatment with 3rd class cephalosporin (Ceftriaxone) and Oxacillin, with outstandingly favorable evolution for ~7 days, with considerable improvement of clinical symptoms and inflammatory biomarkers (no fever, CRP 14.1 mg/L). Nevertheless, on the 8th day of admission the fever reappeared, and thus we repeated the laboratory tests noticing an increase in leukocytes (Leu 33,440/μL) and acute phase reactants levels (CRP 82.96 mg/L, ESR 140 mm/H), and a decrease in hemoglobin (6.6 g/dL), but the peripheral blood cells were normal. We repeated an emergency thoracic radiography which revealed a higher amount of pleural effusion, and thus a surgical drainage was performed inserting a pleural tube for 7 days (Figure 1). The aspect of the pleural fluid was clear, of yellowish color, and both cytology and culture were negative. We ruled out lung tuberculosis based on a negative exam of the fasting gastric fluid obtained by naso-gastric tube, but the pulmonology consult raised the suspicion of methicillin resistant *Staphylococcus aureus* recommending wide-spectrum antibiotics (Meropenem and Vancomycin). A blood transfusion was also administered. After ~5 days of treatment with the antibiotics mentioned above, the fever persisted and we found no improvement in laboratory parameters. Based on the initial outstandingly favorable evolution, we raised the suspicion of a possible superinfection, and we identified positive IgM for both MP and CP (IgM anti-MP 15 Index, IgG anti-MP 0.1 UA/mL, IgM anti-CP 42.3 U/mL, and IgG anti-CP 17.4 U/mL). We established the diagnosis of pneumonia caused by the coinfection with MP and CP, and we changed the antibiotic therapy on Levofloxacin for 10 days. On the 5th day of Levofloxacin we performed a thoracic computed tomography which showed lung window-encapsulated pleural effusion (Figures 2, 3), and we suppressed the thoracic drain tube. We discharged the patient on the 10th day of Levofloxacin with the recommendation to continue the treatment with Clarithromycin orally for 7 days.

### Follow-Up and Outcome

**Figure 4:**
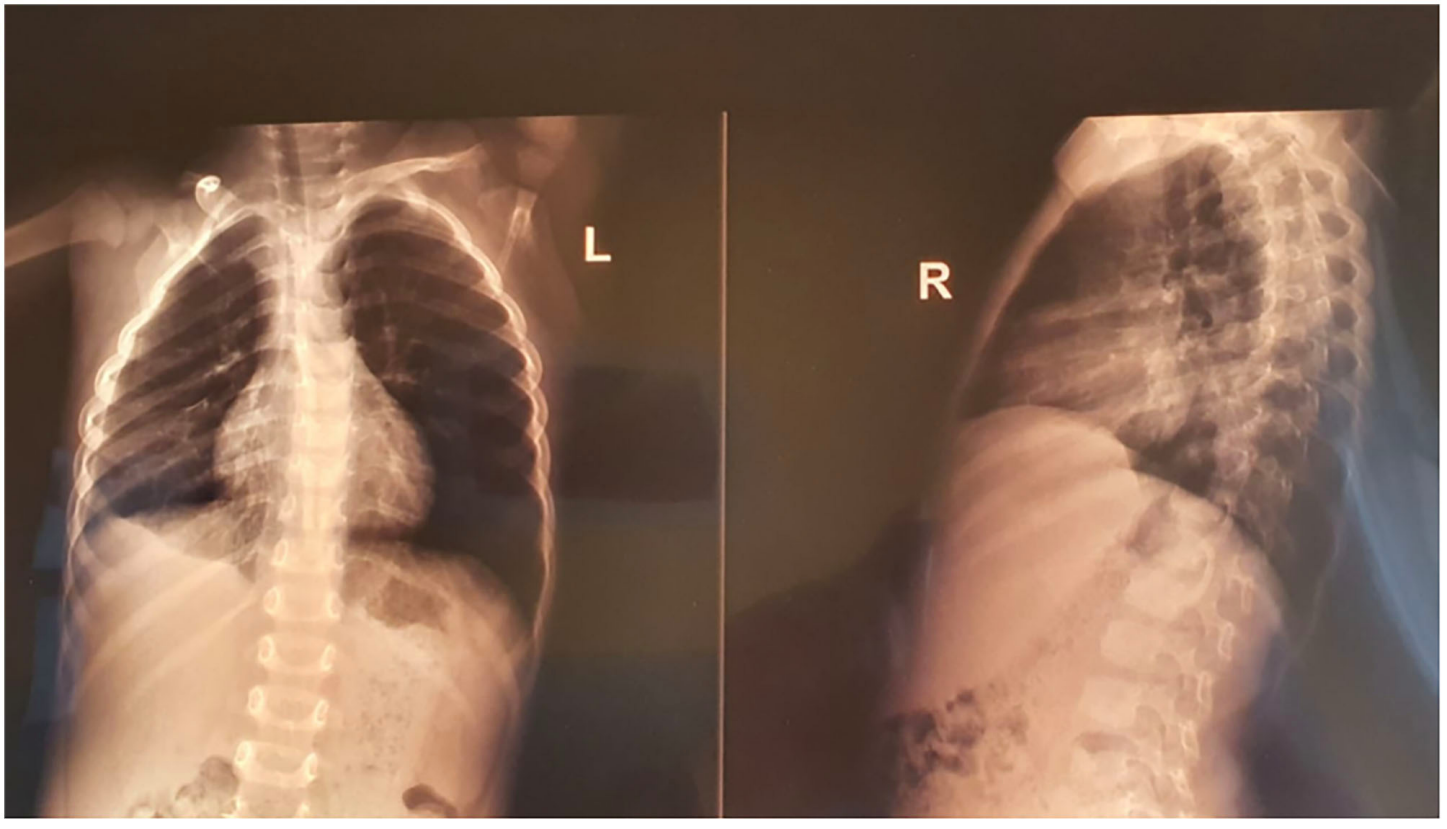
The radiological aspect at the time of discharge.

**Figure 5:**
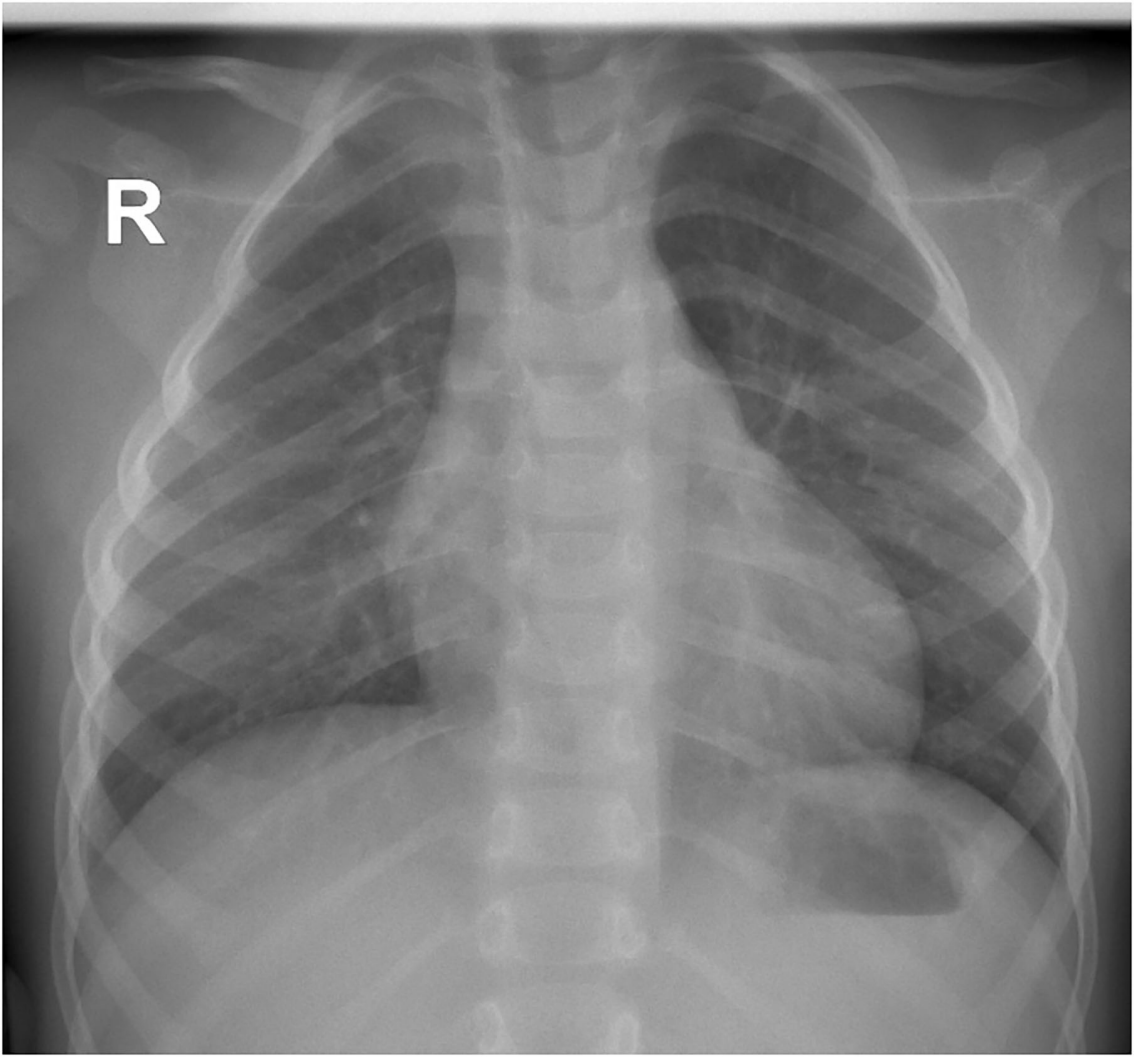
Thoracic radiography after 6 months revealing only minimal thickening of the inferior right pleura.

We repeated the laboratory tests after 7 days of Clarithromycin and they were all within the normal ranges, as well as the thoracic radiography which revealed a diminished quantity of encapsulated pleural effusion (Figure 4). Therefore, we decided to diminish the dose of Clarithromycin at 1/3 of the initial for 1 month. Moreover, after ~1 month from the initial diagnosis, we noticed a seroconversion in terms of IgG for both MP and CP (IgG anti-MP 70 UA/mL, and IgG anti-CP 100 U/mL). The chest radiography performed after ~6 months revealed only a mild thickening of the inferior right pleura (Figure 5).

## Discussions

MP and CP are two atypical pathogens increasingly recognized during the last years for causing community acquired pneumonia in children (5). The prevalence of both pathogens widely varies in children with acute respiratory infections reaching up to 40% (19). Their incubation period was reported to be between 2 and 4 weeks, being transmitted by respiratory route throughout the year (20). The incubation period in our case was more than 2 weeks, though we must also take into account that these two atypical pathogens might have been a cause of superinfection based on the patient's favorable evolution during the 1st week of hospitalization when he was treated with 3rd class cephalosporin and Oxacillin as empirical therapy. Despite possible outbreaks in institutional settings, their rate of transmission is considerably higher between family members or individuals living in close proximity (20). None of the family members or other individuals that closely interacted with our patient presented any symptoms suggesting a possible transmission. A recent study showed that the highest prevalence of both MP and CP was encountered in infants between 29 days and 2 months of life and children between 1 and 5 years (19). Moreover, it was reported that severity varies with age being proved that in case of MP severe community acquired pneumonia occurs around ~21 months, for CP ~49 months, while for co-infection with MP and CP ~24 months (21). Similarly, our patient's age at the time of diagnosis was 33 months. Nevertheless, taking into account the possible self-limiting course of pneumonia caused by these two atypical agents, an explanation of these data might be the fact that usually the studies are performed on hospitalized children that are usually symptomatic. Smoking in house was proved to be a risk factor for respiratory tract infections (14). In our case the patient's mother was a smoker.

The clinical spectrum of MP and CP pneumonia involve cough, fatigue, throat pain and fever in hospitalized patients (14). Moreover, productive cough was reported as the most common symptom in patients with MP infection accounting for ~97% of the cases, while fever was described in 20% of them (22). Our patient presented both productive cough and prolonged fever. Another well-documented fact is that primary infection commonly occurs in younger ages, whereas reinfection is a possibility in older children with the decrement of immunity or depending on genetic susceptibility (14, 23). Primary infection is the most-likely possibility in our case based on both his small age and previously healthy status. Physical examination usually reveals rales on lung fields in case of both MP and CP infections, though they were more frequently noticed in case of MP (14). Our patient's clinical exam at the time of admission also revealed rales on the right lung field. In terms of laboratory tests and imagistic investigations, the findings reported in the literature are controversial. Thus, a study performed by Esposito et al. (24) showed no association between either laboratory parameters nor radiological findings and MP or CP infections. Contrariwise, other studies stated that leukocyte count and ESR were significantly higher especially in acute cases (14). The decrease in hemoglobin is a well-known marker of infection, but it can also occur in case of prolonged inflammation, acute hemorrhage or certain intoxications (9, 25–27). Our patient presented a persistent elevated leukocyte count and an ESR of over 100 mm/h. The initial CRP value was 303 mg/L which considerably decreased after the 1st week of admission. Despite the fact that his condition worsened again after this period, the CRP value was considerably lower as compared to the initial one. The value of hemoglobin decreased during admission requiring blood transfusion. Thus, we might hypothesize again that MP and CP represent coinfections in our case and that the initial condition was caused by a completely different agent, most-likely a β-lactam sensitive one.

A wide-spectrum of radiological findings were reported in patients with community acquired pneumonia caused by atypical bacteria involving interstitial infiltration, reticular aspect, hilary lymphadenopathy, lobar pneumonia, and pleural effusion (14). In our case, the radiological findings consisted in right lobar pneumonia and pleural effusion requiring surgical drainage. Despite the fact that cell culture is approved as a gold standard for the diagnosis of MP and CP infection, it is hindered by their slow-growth and the need for particular growing conditions. Serological tests are a useful diagnostic tool if used appropriately. Thus, a study performed on Indian children underlined that the detection of MP was possible based on positive IgM alone in most of the cases included in the study (28). The diagnosis in our case was also performed on the basis of positive IgM for both MP and CP. Nevertheless, Lee et al. (29) proved that short-term paired IgM-anti MP in the acute phase might be an accurate diagnostic tool for MP pneumonia. Pneumonia caused by atypical pathogen usually require antimicrobial agents especially in children below the age of 5 years due to their complicated course in these group (28). Most-likely the evolution in our case was also hindered by both the small age and presence of coinfection. Another study that included 1,104 Japanese children with acute lower respiratory tract infections identified acute CP infection in 149 of the cases, acute MP infection in 118 children, and the presence of both in only 27 patients (30). Effective management of these types of infections might be achieved either with macrolides or with fluoroquinolones (18, 28). We decided to initiate treatment with fluoroquinolone by vein due to the patient's severe condition, and in agreement with the pediatric surgeon we continued the treatment with macrolide orally based on the persistence of radiological changes.

The pathogenesis of acute lung injury in case of atypical bacteria is not fully understood. Nevertheless, it seems that MP pneumonia fits the PHS hypothesis in which the adaptive immune systems exerts a control on pathogenic or etiological protein substances via a recombination of immune genes for both B and T-cells receptors (31). Moreover, the innate immune system has the ability to control larger substances like bacteria, intact viruses and apoptotic bodies through phagocytes; while toll-like receptors, complements, natural antibodies and other immune protein systems exert their control on smaller non-protein substances among which viral DNAs and RNAs, as well as polysaccharides (31). Thus, MP infection most-likely results in acute organ cell injury, which will further result in hyperimmune reactions and consequently a critical immune-mediated lung injury similar to COVID-19 (31). Most-likely our patient also developed a hyperimmune response to this infection, further augmented by the prior or simultaneous presence of CP. Based on the above-mentioned hypothesis, the use of corticosteroids in these cases might be benefic taking into account their role in inducing early stabilization of hyperimmune responses by influencing the host immune system (31). The utility of corticosteroids during the early phase of MP pneumonia was further sustained by Yang et al. (4) on a study on 257 children proving that it might be associated with lower morbidity, preventing the progression of this condition. Our patients received corticosteroids during the first 5 days of admission taking into account the low oxygen saturation, indeed with outstandingly favorable evolution fact that suggest once more most-likely the presence of another pathogen initially.

The limitations of this case report consist of a relative delay in terms of diagnosis, but acknowledging and reporting the challenges related to it might be considered highly important in daily practice and they might improve the pediatrician's awareness regarding the peculiarities of these atypical bacteria in small children. Another important limitation is represented by the fact that we were not able to perform other diagnostic tools besides serology, such as polymerase chain reaction-based test or culture, but taking into account the initial seropositivity of both IgM anti-MP and Ig-M anti-CP associated with negative IgGs for both bacteria, the outstandingly favorable evolution after the initiation of Levofloxacin, and the further seroconversion of IgGs for both atypical agents, we consider that we are entitled to sustain our diagnosis.

It is also important to acknowledge another possible circumstance consisting in the fact that our patient might have experience a recent prior infection with CP being further infected with MP and developing a severe form of MP pneumonia. The recent infection with CP might have triggered the abnormal immune response in terms of MP infection resulting in severe lung injuries. Thus, both simultaneous co-infection and prior infection with CP and further one with MP might result in the same life-threatening severe form of atypical pneumonia as in our patient, who to the best of our knowledge it is the first case reported in Romania with severe community acquired pneumonia due to the interaction between MP and CP.

## Conclusions

MP and CP are two atypical pathogen that are difficult to be diagnosed due to their slow-growing pattern. Despite their self-limiting feature, the association between them might carry a vital risk in small children, especially in the lack of a proper and timely diagnosis.

## Data Availability Statement

The original contributions presented in the study are included in the article/supplementary material, further inquiries can be directed to the corresponding author/s.

## Ethics Statement

Written informed consent was obtained from the patient's mother prior to publication of this case.

## Author Contributions

CM, LM, and MS conceptualized and designed the study, drafted the initial manuscript, and reviewed and revised the manuscript. MS and IS designed the data collection instruments, collected data, carried out the initial analyses, and reviewed and revised the manuscript. All authors approved the final manuscript as submitted and agree to be accountable for all aspects of the work.

## Conflict of Interest

The authors declare that the research was conducted in the absence of any commercial or financial relationships that could be construed as a potential conflict of interest.
